# Investigations on Binding Pattern of Kinase Inhibitors with PPAR*γ*: Molecular Docking, Molecular Dynamic Simulations, and Free Energy Calculation Studies

**DOI:** 10.1155/2017/6397836

**Published:** 2017-02-22

**Authors:** Mohit Mazumder, Prija Ponnan, Umashankar Das, Samudrala Gourinath, Haseeb Ahmad Khan, Jian Yang, Meena Kishore Sakharkar

**Affiliations:** ^1^Structural Biology Laboratory, School of Life Sciences, Jawaharlal Nehru University, New Delhi, India; ^2^Drug Discovery and Development Research Group, College of Pharmacy and Nutrition, University of Saskatchewan, 107 Wiggins Road, Saskatoon, SK, Canada S7N 5C9; ^3^Department of Biochemistry, College of Science, King Saud University, Riyadh, Saudi Arabia

## Abstract

Peroxisome proliferator-activated receptor gamma (PPAR*γ*) is a potential target for the treatment of several disorders. In view of several FDA approved kinase inhibitors, in the current study, we have investigated the interaction of selected kinase inhibitors with PPAR*γ* using computational modeling, docking, and molecular dynamics simulations (MDS). The docked conformations and MDS studies suggest that the selected KIs interact with PPAR*γ* in the ligand binding domain (LBD) with high positive predictive values. Hence, we have for the first time shown the plausible binding of KIs in the PPAR*γ* ligand binding site. The results obtained from these in silico investigations warrant further evaluation of kinase inhibitors as PPAR*γ* ligands in vitro and in vivo.

## 1. Introduction

Peroxisome proliferator-activated receptors (PPARs) belong to the nuclear receptor super family and are ligand activated transcription factors, regulating the expression of a wide variety of genes. On activation by a ligand, they bind to the PPAR-responsive regulatory elements (PPRE) and/or PPAR associated conserved motif (PACM) as obligate heterodimers with retinoid X receptor (RXR) [1, 2]. Similar to other nuclear receptor-family members, PPARs are multidomain proteins, consisting of an N-terminal transactivation domain (AF1), a highly conserved DNA-binding domain (DBD), and a C-terminal ligand binding domain (LBD) which has a ligand-dependent transactivation function (AF2) [3, 4]. Three isoforms of PPARs (alpha, beta/delta, and gamma) have been identified so far in human, mouse, rats, xenopus, and hamsters [5–7] and among them, PPAR*γ* is the most intensively studied. PPAR*γ* has three alternatively spliced isoforms and all of them are expressed in adipose tissues [8, 9]. It is primarily involved in the regulation of lipid metabolism and insulin sensitivity reactions and also plays an important role in carcinogenesis and cell physiology [10, 11]. Also, PPARs have been shown to have ligand independent repression whereby they repress the transcription of direct target genes by recruitment of corepressor complexes which blocks the actions of coactivator complexes [12]. PPAR*γ* activation is involved in transcriptional regulation of genes involved in proliferation, angiogenesis, apoptosis, organogenesis, and energy metabolism and hence implicated in cell growth and viability [13–16]. PPAR*γ* signaling is modulated using different domains and various natural lipophilic agonists (ligands) such as unsaturated fatty acids, oxidized lipid species, eicosanoids, and prostaglandins [2, 17, 18]. Conformational changes caused by ligand binding lead to the modulation of PPAR*γ* activity by differential recruitment of cofactors [4, 12]. PPAR*γ* exhibits high affinity towards thiazolidinediones (TZDs) [19]. TZDs including troglitazone, rosiglitazone, and pioglitazone are FDA approved synthetic agonists of PPAR*γ* [20, 21]. TZDs bind to the LBD of PPAR*γ*, activating the AF2 surface to accommodate the coactivators. The LBD of PPAR*γ* is largely a helical domain comprising 13 *α*-helices (H1−H12, H2′) and one parallel *β*-sheet. The AF2 region (ligand-dependent transactivation function) is formed by helices 11 and 12 and consists of hydrophobic residues [22]. However, the use of TZDs has recently been restricted due to various side effects that include renal fluid retention, hemodilution, weight gain, edema, cardiomegaly and congestive heart failure, and loss of bone mineral density [23, 24]. In view of the above reported undesirable side effects at therapeutic dosages, the focus has shifted to the discovery, development, and evaluation of “selective PPAR*γ* modulators” or SPPARMs as safer alternatives to PPAR*γ* full agonists. In contrast to the full agonists, the partial agonists show reduced transcriptional activity while having retained the insulin sensitization and hence show promising therapeutic potential with fewer side effects in animal models [25, 26]. The acidic thiazolidinedione moiety of full agonists such as rosiglitazone forms strong hydrogen bonding network with the side chains of His323, His449, and Tyr473 from helices 5, 7, and 12, respectively, of PPAR*γ* and stabilizes AF2 to recruit coactivators [22]. However, partial agonists tend to stabilize the *β*-sheet region by acidic substituents or form hydrophobic interactions and do not stabilize helix H12 via hydrogen bonding with Tyr473 [27, 28].

On a separate note, PPAR*γ* undergoes several posttranslational modifications including phosphorylation of Ser273 by extracellular signal-regulated kinase ERK/cyclin-dependent kinase 5 (Cdk5) [29, 30]. Moreover, the underlying mode of action for both full agonist and partial agonists to elicit antidiabetic property involves the inhibition of obesity-linked phosphorylation of Ser273 in PPAR*γ*. Hence rather than transactivation of the genes, the agonists cause conformational change in the LBD of PPAR*γ* preventing the kinase to phosphorylate the serine residue [29, 30].

**Figure 1:**
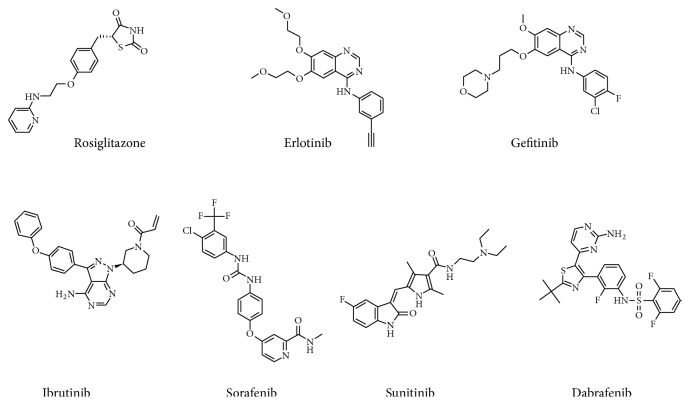
Structures of PPAR*γ* full-agonist rosiglitazone and FDA approved kinase inhibitors.

**Figure 2:**
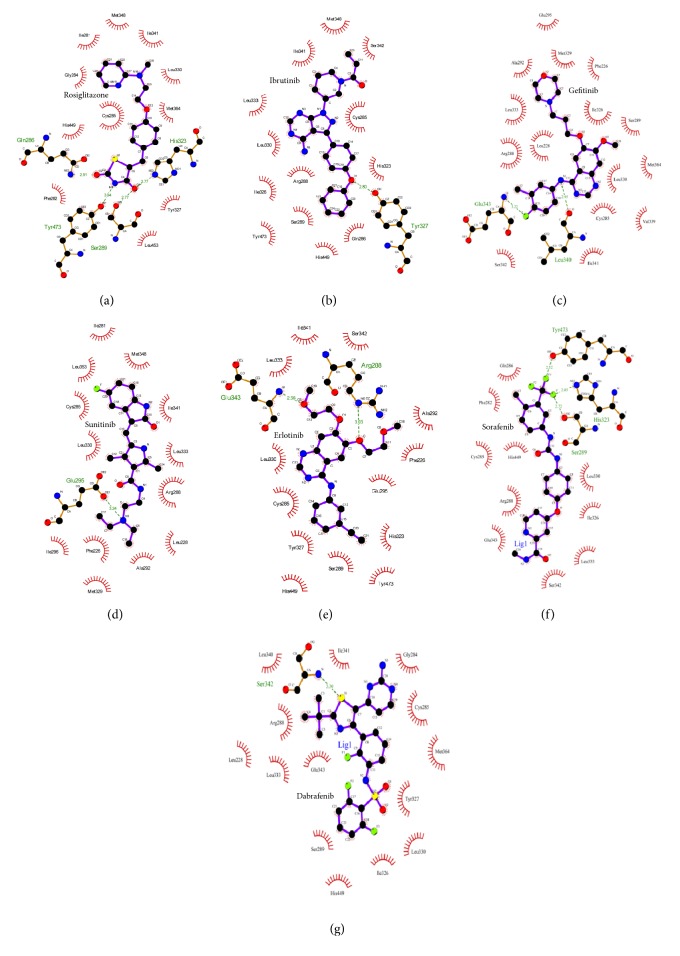
2D-docking pose showing KIs and rosiglitazone in the binding site of human PPAR*γ* using LigPlot software. The interactions shown are those mediated by hydrogen bonds and by hydrophobic contacts. Hydrogen bonds are indicated by dashed lines between the atoms involved, while hydrophobic contacts are represented by an arc with spokes radiating towards the ligand atoms they contact. The contacted atoms are shown with spokes radiating back.

The associations of PPAR*γ* with signaling molecules including receptor and nonreceptor kinases corroborate the cross-talk function between the two signaling proteins [11, 24, 25, 30, 31]. Kumar et al. (2005) identified L-tyrosine derivatives as potential PPAR*α*/*γ* inhibitors [32, 33]. De Filippis et al. described the synthesis and the evaluation of PPAR activity of the new tyrosine derivatives, based on the combination of GW409544, a potent full agonist on both PPAR*α* and PPAR*γ* and stilbene or phenyldiazene scaffolds [34]. Interestingly, some known ligands of PPAR*γ* (e.g., Honokiol, amorfrutin 1, amorphastilbol, and hydroxyhydroquinone) have tyrosine moiety like substructure and structural similarity to tyrosine kinase inhibitors. This led us to speculate on the possibility of TKI being ligands for PPAR*γ*. Structural superimposition of synthetic ligand rosiglitazone and selected TKI further confirmed our speculation. A question was posed whether these kinase inhibitors activate PPAR*γ* and could be potential PPAR*γ* ligands. The objectives of the present study were to (1) investigate the interaction of selected kinase inhibitors such as ibrutinib, sorafenib, sunitinib, erlotinib, gefitinib, and dabrafenib with PPAR*γ* in silico (Figure 1), (2) a comparative analysis of interaction of these KIs with rosiglitazone in the LBD of human PPAR*γ* using molecular docking studies, and (3) molecular dynamic simulation and MM/PB (GB) SA studies to evaluate the stability and conformational changes due to the interaction of the kinase inhibitors in the PPAR*γ* binding site.

## 2. Materials and Methods

### 2.1. Protein and Ligand Preparation

The starting structure for the simulations was taken from the X-ray structure of the ligand binding domain and coactivator assembly of PPAR*γ* (PDB code 2PRG, resolution: 2.3 Å) [21]. The protein was prepared using Schrodinger's protein preparation wizard [35] by removal of crystallographic water molecules and addition of hydrogen atoms, followed by minimization and optimization using OPLS2005 force field of Schrodinger [36]. The shape and properties of the receptor were represented on a grid by several different sets of fields that provide progressively more accurate scoring of the ligand poses. The SDF files for the drugs rosiglitazone, ibrutinib, sorafenib, sunitinib, erlotinib, dabrafenib, and gefitinib were obtained from PubChem database (https://pubchem.ncbi.nlm.nih.gov/). These molecules were then prepared in Schrodinger Ligprep wizard. During the ligand preparation all possible conformations were taken into account. The ligands were subjected to further predocking preparations where hydrogens were added followed by minimization and optimization in OPLS_2005 force field as implemented on Maestro software. Finally, 32 conformations of each ligand were generated and used for docking.

### 2.2. Docking of Ligands in PPAR*γ* Binding Domain Using Glide

Molecular docking procedures were carried out after preparing the ligand library using Schrodinger Ligprep module and defining the grid corresponding to the ligand binding site of the protein. The grid was prepared using rosiglitazone at the center and the interacting residues as the ligand binding site residues along with a cubic space of 12 angstroms around the ligand rosiglitazone. The Glide module of Schrodinger uses Systematic and Simulation Method for searching flexible ligand poses. In a systematic method, it uses incremental construction for searching, and its output *G*-Score is an empirical scoring function which is a combination of various parameters.

### 2.3. Molecular Dynamics Simulations

The poses with highest Glide scores obtained from the docking simulations (protein-ligand complexes) were further subjected to MD simulations. The purpose of performing the MD simulations was to determine the stability of the drug molecules and to understand the binding pattern and to identify the binding residues in the receptor to the drug molecule. Parameters for all the drug molecules (bound at the ligand binding site) were generated using antechamber module of AMBER suite [37, 38]. The restrained electrostatic potential (RESP) was used to describe the partial atomic charges. Then the general AMBER force field (GAFF) [39] was used to describe the parameters of drug molecules. The standard AMBER force field for bioorganic systems (ff99SB) was employed to describe the protein, followed by the addition of hydrogen atoms and counterions to neutralize the system. The input files for energy minimization, dynamics, and analysis were prepared with xleap. Both systems were solvated using atomistic TIP3P water in a box with edges at least 12 Å from the complex.

All simulations were performed using AMBER molecular dynamics suite version [37]. Energy minimization was first conducted with the steepest descent method and then switched to conjugate gradient every 500 steps for a total of 5000 steps with 0.1 kcal/mol Å^2^ restraints on all atoms of the complexes. Following this step, another two rounds of energy minimization were performed by only restraining the protein and further releasing all the restraints for 2000 steps of each round. Long-range Coulombic interactions were handled using the particle mesh Ewald (PME) summation. For the equilibration and subsequent production runs, the SHAKE algorithm was employed on all atoms covalently bonded to a hydrogen atom, allowing for an integration time step of 2 fs. The system was gently annealed from 0 K to 300 K over a period of 50 ps using a Langevin thermostat with a coupling coefficient of 1.0 ps and 50 ps of density equilibration with weak restraints. The system was again equilibrated for 500 ps without any restraints. The production phase of the simulations was run without any restraints for a total of 25 ns on each system. Coordinates and energy values were collected every 10 ps throughout the simulations.

### 2.4. Binding Free Energy Calculations

The binding free energies of PPAR*γ* for all the KI molecules were analyzed by the MM/PB (GB) SA scripts, integrated in the AMBER 12 software package. In this procedure, snapshots were first extracted from the obtained trajectories. For each snapshot, free energy is calculated for the protein, ligand, and complex using single trajectory approach. The binding free energy was computed as the difference:

(1) $$\begin{array}{c} \Delta G_{\text{bind}}=G_{\text{complex}}-G_{\text{protein}}-G_{\text{ligand}}. \end{array}$$

### 2.5. Per Residue Interaction Decomposition

To determine the contribution of each residue to the binding energy, the MM-GBSA method was used. MM-GBSA method decomposes the interaction energies for each residue by considering molecular mechanics and solvation energies without consideration of the contribution of entropies. Each residue contribution includes three terms: van der Waals contribution (Δ*G*_vdw_), electrostatic contribution (Δ*G*_ele_) in a vacuum, and solvation contribution (Δ*G*_solvation_).

(2) $$\begin{array}{c} \Delta G\text{ residue}=\Delta G_{\text{vdw}}+\Delta G_{\text{ele}}+\Delta G_{\text{solvation}}. \end{array}$$

All energy components in the above equation were calculated using 1000 snapshots from the last 10 ns of the MD simulation. The calculations and the computational methods used in this paper are well documented in the literature and have been used previously for small molecules [40].

## 3. Results and Discussion

### 3.1. Molecular Docking Studies

**Figure 3:**
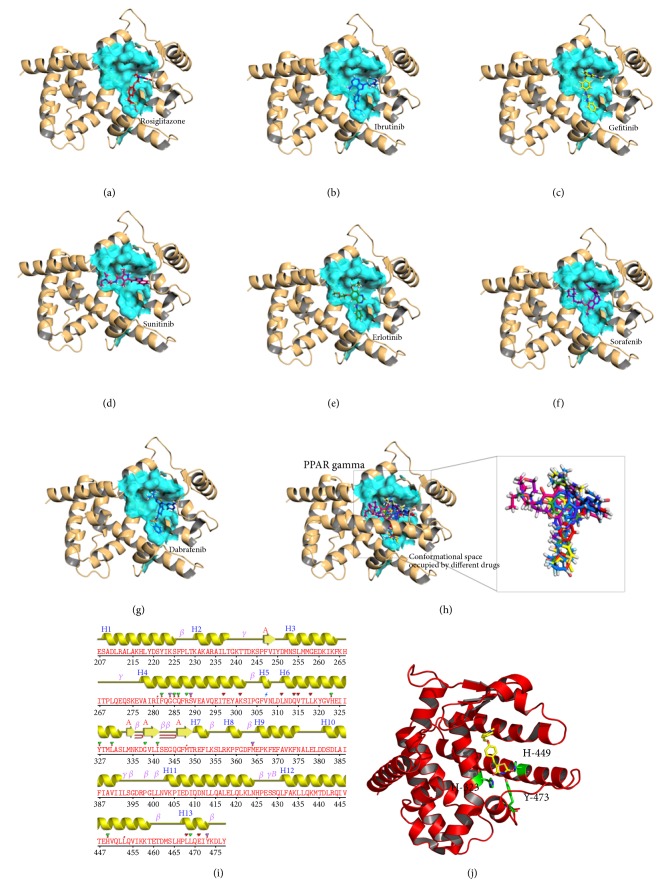
Postdocking interactions between active site residues of PPAR gamma with ligand ((a) rosiglitazone, (b) ibrutinib, (c) sorafenib, (d) sunitinib, (e) erlotinib, (f) gefitinib, and (g) dabrafenib). (h) Binding from all the poses obtained from all the drugs used in this study. The protein is depicted in cartoon and surface representation view and ligands as sticks in the binding pocket. (i) Secondary structure of 2PRG with amino acid residues mapped obtained using PDBSum program. (j) Crystal structure of PPAR gamma with rosiglitazone showing the position of His323, His449, and Tyr473 from helices 5, 7, and 12, respectively.

A ligand is stabilized energetically at the binding site in the protein structure by weak intermolecular interactions such as hydrogen bonds and hydrophobic interactions [31]. In the present study, the binding mode for KIs in the ligand binding site of human PPAR*γ* was investigated using molecular docking studies. The docking results and the docked conformations of KIs are illustrated in Table 1 and Figures 2 and 3. These docking results clearly indicate that the KIs used in the study exhibit significant binding affinities towards human PPAR*γ* protein (Glide XP score range −10.50 kcalmol^−1^ to −7.75 kcalmol^−1^) and the energy ranges (Glide energy range −59.65 kcalmol^−1^ to −46.71 kcalmol^−1^) are comparable to the cocrystalized molecule, rosiglitazone (2PRG) (Table 1). Figures 2 and 3 illustrate the binding pose of KIs and rosiglitazone in the binding pocket of human PPAR*γ* protein, respectively. Ibrutinib with a lower binding energy of −59.65 kcalmol^−1^ and considerably good Glide XP score of −10.50 kcalmol^−1^ (Table 1) forms H-bond interaction between the oxygen atom of diphenyl ether side chain and the phenolic hydroxyl group of Tyr327 (helix 5) of protein (Figure 2(b)). The interaction of ibrutinib is stabilized by hydrophobic interactions with *β*-sheet and turn residues Ile341, Ser342, and Met348 (Table 1, Figure 2(b)). Molecular overlay of binding pose for ibrutinib/PPAR*γ* in the surface volume of rosiglitazone/PPAR*γ* indicates that the conformation of ibrutinib in the PPAR*γ* binding pocket is similar to that of rosiglitazone (Figure 3(h)). Gefitinib/PPAR*γ* docked complex with Glide XP score −9.10 kcalmol^−1^ and Glide energy −52.45 kcalmol^−1^ shows H-bond interactions between fluorine substituent of 3-chloro-4-fluorophenyl group of the ligand and main chain nitrogen atom of Glu343 (*β*-turn) of the protein. Gefitinib forms another H-bond interaction between NH linker connecting the quinazoline ring and 3-chloro-4-fluorophenyl ring and the main chain carbonyl oxygen atom of Leu340 of PPAR*γ* (Figure 2(c)). The docking conformation of gefitinib in the PPAR*γ* binding site is stabilized by hydrophobic interactions (Table 1, Figure 2(c)). The orientation of gefitinib in the PPAR*γ* binding pocket is more towards the *β*-sheet region (Figure 3(c)).  Figure 2(d) and Table 1 reveal that sunitinib forms a very stable sunitinib/PPAR*γ* complex (Glide XP score = −7.75 kcalmol^−1^ and Glide energy = −46.71 kcalmol^−1^) by forming single H-bond interaction with nitrogen atom of terminal diethyl amine group and side chain oxygen atom of Glu295 (helix H3). Further, the binding pose of sunitinib/PPAR*γ* complex shows that sunitinib does not overlay over the conformation of rosiglitazone in the PPAR*γ* binding pocket, rather sunitinib is more inclined to helixes H3 and H5 (Figure 3(d)). The other KI, erlotinib, forms H-bond interactions with Arg288 of helix H3 and Glu343 of *β*-turn, through the oxygen atoms of its two terminal 2-methoxyethoxy groups. The ethyloxy oxygen atom of 2-methoxyethyloxy side chain of erlotinib forms H-bond with main chain amino nitrogen atom of Arg288. Main chain nitrogen atom of Glu343 forms H-bond interaction with side chain methoxy oxygen atom (Figure 2(e)). The fluorine atom of trifluoromethyl substituent of sorafenib forms three H-bond interactions. Side chain oxygen atom of Tyr473, *ε*-nitrogen of imidazole ring of His323, and side chain oxygen atom of Ser289 are the contributors to the H-bond formation with sorafenib (Figures 2(f) and 3(f)). Dabrafenib forms a single H-bond interaction between sulfur atom of 3-butyl thiazolyl ring and the main chain nitrogen atom of Ser342. The interaction patterns for KI/PPAR*γ* as observed from molecular docking studies suggest KIs as PPAR*γ* ligands.

### 3.2. Molecular Dynamics Simulation Studies of the PPAR*γ*-KI Complexes

**Figure 4:**
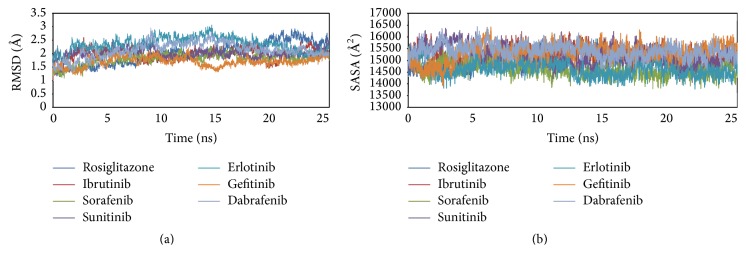
Molecular dynamics simulation of PPAR*γ*-ligand complexes. (a) RMSD of human PPAR*γ* backbone structure docked with KIs and cocrystalized compounds rosiglitazone in 25 ns simulation. (b) SASA of human PPAR*γ*-KI and of human PPAR*γ*-rosiglitazone complexes in 25 ns simulation.

MD simulations for each protein-KI complex and the standard complex of protein-rosiglitazone were performed for 25 ns with 500 ps equilibration and 10 ps of data collection for each complex (Figure 4). MD simulation is one of the useful tools to investigate the stability of the protein-ligand complexes in different docking poses under different physiological conditions. In order to evaluate the possible deviation in the structure during simulation, RMSD were calculated based on the initial backbone coordinates of the protein-ligand complexes. The plot for RMSD indicates that stability in the interactions for all the protein-ligand complexes is attained after 15 ns of simulation. The most stable interaction was observed for PPAR*γ*-gefitinib complex as it reached the equilibrium faster with lowest RMSD level. The curves for PPAR*γ*-sorafenib complex were quite similar to the standard PPAR*γ*-rosiglitazone, showing stable RMSD after 10 ns. PPAR*γ*-ibrutinib complex shows a high RMSD value initially attains stable conformation after 10 ns at a slightly higher level than PPAR*γ*-rosiglitazone complex. This is followed by PPAR*γ*-dabrafenib and PPAR*γ*-erlotinib complexes that have highest initial RMSD values and attain stability after 15 ns. Moreover, after 15 ns the average RMSD for all the complexes was approximately 1.5 Å to 2.5 Å and remains stable throughout the 25 ns simulation. However, it is interesting to observe that there is a sudden decrease in the stability of PPAR*γ*-rosiglitazone complex around 20 ns with high value of RMSD (2.5 Å) for the complex. The results from the RMSD plot indicate that the PPAR*γ*-KI and the standard PPAR*γ*-rosiglitazone systems could be satisfactorily explored in the allocated nanosecond simulation studies.

The solvent accessible surface area (SASA) plot for PPAR*γ*-KI and the standard PPAR*γ*-rosiglitazone complexes show no significant changes in the equilibration curve throughout 20 ns MD simulation run (Figure 4(b)). This indicates that surface of all the protein-ligand complexes has maintained a similar accessibility to the solvents.

**Figure 5:**
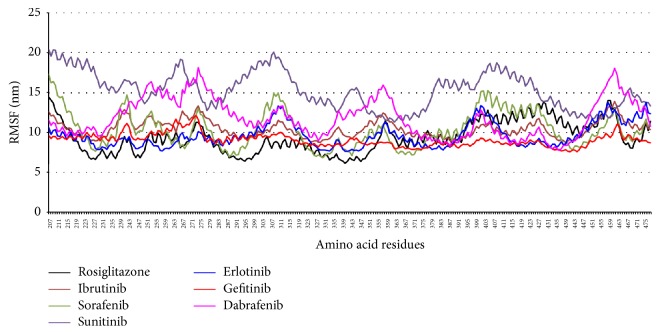
RMSFs for residues 207–476 of human PPAR*γ* complexes with KIs and rosiglitazone at 25 ns molecular dynamics simulation.

Plot for root mean square fluctuations (RMSFs) (Figure 5) represents the stability of each residue in the protein-ligand complexes over 25 ns MD simulation. The RMSF curve for the docked poses containing PPAR*γ*-KI complexes with sorafenib, ibrutinib, erlotinib, and gefitinib shows little fluctuation (~10 nm), similar to the standard PPAR*γ*-rosiglitazone complex. The most unstable complexes PPAR-sunitinib and PPAR*γ*-dabrafenib show very high values of fluctuations. PPAR*γ*-sunitinib complex shows the highest value of fluctuation in RMSF for Glu207, Lys261, Val315, and Leu414. Moreover, PPAR*γ*-dabrafenib complex shows high RMSF value for Met252, Gln279, Phe360, and Glu460. Nonetheless, structural stability is observed for all the PPAR*γ*-ligand complexes around the ligand binding site residues including Cys285, Gln286, Ser289, His323, Leu330, Met348, and Tyr473.

### 3.3. Binding Free Energy Analysis by MM-PBSA and MM-GBSA

The common and popular approaches to estimate the free energy of the binding of small ligands to biological macromolecules are the molecular mechanics energies combined with the Poisson–Boltzmann or Generalized Born and Surface Area continuum solvation (MM/PBSA and MM/GBSA) [41]. One of the limitations in most of the scoring functions is the handling of solvent effects. This problem could be solved using scoring functions involving physical approximations like MM-PBSA and MM-GBSA. Despite containing several approximations, MM/PBSA and MM/GBSA approaches have helped validate and rationalize experimental findings and improve the results of virtual screening and docking [41]. The main objective of this method is to find the difference in the free energies of bound and unbound state of protein-ligand complexes. All the thermochemical properties were calculated by MM-PBSA and MM-GBSA approach using AMBER program, for each coordinate at every 10 ps sampling frequency throughout the MD trajectory for all the protein-ligand complexes. The complexes with lowest binding energy are considered to be most stable (Table 2).

The total free energies (Δ*G*_TOT_) obtained from MM-GBSA for the protein-ligand complexes show comparable values ranging from −49.82 kcalmol^−1^ to −33.10 kcalmol^−1^ (−49.82 kcalmol^−1^ for PPAR*γ*-erlotinib complex and −33.10 kcalmol^−1^ for PPAR*γ*-sorafenib complex) (Table 2). Van der Waal's energy (Δ*E*_VDW_), electrostatic energy (Δ*E*_ELE_), nonpolar contribution (Δ*E*_NPOL_), and total energy of solute (Δ*E*_GAS_) have negative values and show favorable contribution to the total free energy. However, total energy of solvation (Δ*E*_SOLV_) and polar solvation contribution (Δ*E*_POL(GB)_) possess positive values and therefore contribute unfavorably towards the total free energy.

Table 3 shows total free energies (Δ*G*_TOT_) obtained from MM-PBSA approach for the protein-ligand complexes. PPAR*γ*-erlotinib complex appears to be the most stable complex having a binding energy value of −72.13 kcalmol^−1^, followed by PPAR*γ*-ibrutinib complex (−62.62 kcalmol^−1^). PPAR*γ*-gefitinib complex possesses the lowest free energy value (−39.54 kcalmol^−1^).

Further, the entropy contribution (Δ*S*) was calculated using quasi-harmonic approximation (Table 4). The values for entropy contribution were very similar, indicating no clear involvement of this term in the determination of free energy. Here it is important to mention that it has been suggested that “entropy contributions can be neglected if only a comparison of states of similar entropy is desired such as two ligands binding to the same protein” [42]. Hence the entropy calculations can be ignored.

### 3.4. Individual Residue Contribution in Ligand Binding

**Figure 6:**
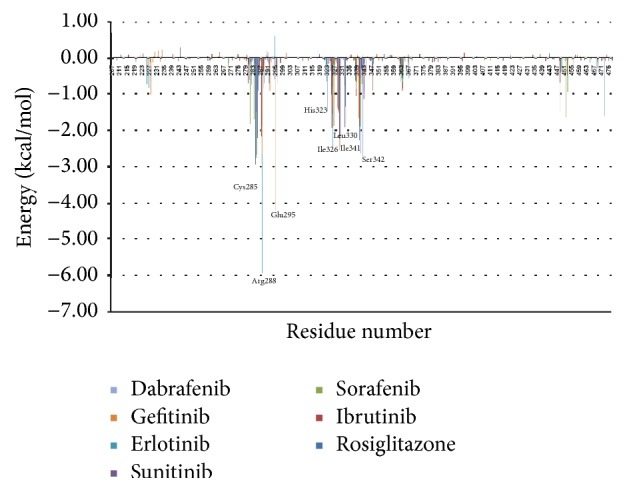
Energy contribution for individual residues 207–476 of human PPAR*γ* complexes with KIs and rosiglitazone to the overall free energy at 25 ns MD simulation.

In order to identify important residues for ligand binding, we calculated the contribution of each individual residue of PPAR*γ* binding with each ligand (Figure 6). For this purpose, Generalized Born (GB) model was used to efficiently compute solvation free energy. The individual residue contributions to the binding free energy vary in the range of +1.0 to −10.0 kcal/mol. Among these 270 residues in the crystal structure of PPAR*γ* (PDB ID: 2PRG), seven residues (Cys285, Arg288, Glu295, Ile326, Leu330, Ile341, and Ser342) have binding free energy contribution (Δ*G*_res_) more than −2.0 kcal/mol (Figure 6). These residues are thus predicted as energetically important for ligand binding inside the ligand binding site via hydrophobic or hydrogen bond interactions in almost all complexes.

## 4. Conclusions

In the present study, we for the first time investigate the role of kinase inhibitors (KIs) as PPAR*γ* ligands. The molecular docking studies show that KIs could bind effectively in the PPAR*γ* pocket by forming important H-bond contacts and hydrophobic interactions, similar to known agonists of PPAR*γ*. The stability of interaction of KIs and PPAR*γ* ligand binding site residues predicted by docking experiments was further assessed by molecular simulation studies. Further, the free energy and entropy calculations provide insight into the binding affinity of KIs towards PPAR*γ*. Overall, we propose KI as promising lead compounds that could be PPAR*γ* ligands. We expect that the structural insights obtained in this study will facilitate the design of novel KI based PPAR gamma ligands. More elaborate studies are needed for validation of these predictions and better understanding of the mechanism of the interaction of KIs with PPAR*γ* and the future clinical applications of KIs.

## Figures and Tables

**Table 1:** Glide docking scores (kcal mol^−1^), docking energies, and calculated hydrogen bond and hydrophobic interactions of TKIs and reference compound rosiglitazone bound to human PPAR gamma binding site.

Compounds	Glide XP *G*-Score	Glide energy	Hydrogen bond (up to 3.6 Å)	Hydrophobic interactions
Ibrutinib	−10.50	−59.65	Tyr-327	His-323, Cys-256, Ser-342, Met-348, Ile-341, Leu-333, Leu-330, Arg-288, Ile-326, Tyr-473, Ser-289, His-449, Gln-289
Sorafenib	−10.49	−53.53	Ser-289, His-323, Tyr-473	Gln-286, Phe-282, Cys-285, His-449, Arg-288, Gln-343, Ser-342, Leu-333, Ile-326, Leu-330
Sunitinib	−7.75	−46.71	Glu-295	Ile-296, Phe-226, Ala-292, Met-329, Leu-228, Arg-288, Leu-333, Ile-341, Met-348, Ile-281, Leu-353, Cys-285, Leu-330
Erlotinib	−9.54	−52.56	Arg-288, Glu-343	Leu-333, Ile-341, Ser-342, Ala-292, Phe-226, Glu-295, His-323, Tyr-473, Ser-289, Tyr-327, His-449, Cys-285, Leu-330
Gefitinib	−9.10	−52.45	Leu-340, Glu-343	Ser-342, Ile-341, Val-339, Cys-285, Leu-330, Met-364, Ser-289, Ile-326, Phe-226, Met-329, Glu-295, Ala-292, Leu-333, Arg-288, Leu-228
Dabrafenib	−8.59	−50.27	Ser-342	Ser-289, His-449, Ile-326, Leu-330, Tyr-327, Met-364, Cys-285, Gly-284, Ile-341, Leu-340, Arg-288, Leu-228, Leu-333, Glu-343
Rosiglitazone	−11.28	−60.57	Gln-286, Ser-289, His-323, Tyr-473	Ile-281, Phe-282, Gly-284, Cys-285, Tyr-327, Leu-330, Ile-341, Met-348, Met-384, Leu-453, His-449

**Table 2:** Calculated total binding energies for PPAR gamma-TKI's and PPAR gamma-rosiglitazone complexes by MM-GBSA.

Energies	PPAR gamma-rosiglitazone (kcal mol^−1^)	PPAR gamma-ibrutinib (kcal mol^−1^)	PPAR gamma-sorafenib (kcal mol^−1^)	PPAR gamma-sunitinib (kcal mol^−1^)	PPAR gamma-erlotinib (kcal mol^−1^)	PPAR gamma-gefitinib (kcal mol^−1^)	PPAR gamma-dabrafenib (kcal mol^−1^)
Δ*E*_VDW_	−49.84	−59.35	−52.24	−46.70	−59.84	−63.12	−57.95
Δ*E*_ELE_	−19.43	−8.70	−17.94	−120.71	−25.86	−102.38	−2.53
Δ*E*_POL(GB)_	35.22	33.21	44.41	127.18	43.51	126.65	33.67
Δ*E*_NPOL_	−6.49	−7.76	−7.33	−7.05	−7.63	−7.57	−7.28
Δ*E*_GAS_	−69.27	−68.05	−70.18	−167.41	−85.70	−165.50	−60.49
Δ*E*_SOLV_	28.73	25.45	37.08	120.13	35.88	119.08	26.39
Δ*G*_TOT_	−**40.54**	−**42.60**	−**33.10**	−**47.28**	−**49.82**	−**46.42**	−**34.10**

Absolute free energy Δ*G*_TOT_ = (Δ*E*_GAS_ + Δ*E*_SOLV_) − *T*Δ*S*; Δ*E*_GAS_ + Δ*E*_SOLV_ = enthalpy; *T*Δ*S* = solute entropy; Δ*E*_GAS_ = total energy of solute, Δ*E*_GAS_ = Δ*E*_VDW_ + Δ*E*_ELE_; Δ*E*_VDW_ = van der Waal's energy; Δ*E*_ELE_ = electrostatic/coulombic energy; Δ*E*_SOLV_ = total energy of solvation; Δ*E*_SOLV_ = Δ*E*_POL(GB)_ + Δ*E*_NPOL_; Δ*E*_POL(GB)_ = polar solvation contribution, generalized Born method; Δ*E*_NPOL_ = nonpolar contribution.

**Table 3:** Calculated total binding energies for PPAR gamma-TKI's and PPAR gamma-rosiglitazone complexes by MM-PBSA.

Energies	PPAR gamma-rosiglitazone (kcal mol^−1^)	PPAR gamma-ibrutinib (kcal mol^−1^)	PPAR gamma-sorafenib (kcal mol^−1^)	PPAR gamma-sunitinib (kcal mol^−1^)	PPAR gamma-erlotinib (kcal mol^−1^)	PPAR gamma-gefitinib (kcal mol^−1^)	PPAR gamma-dabrafenib (kcal mol^−1^)
Δ*E*_VDW_	−49.84	−59.35	−52.24	−46.70	−59.84	−63.12	−57.96
Δ*E*_ELE_	−19.43	−8.70	−17.94	−120.71	−25.86	−102.38	−2.54
Δ*E*_POL(GB)_	45.87	47.40	61.60	158.29	55.93	168.21	48.17
Δ*E*_NPOL_	−34.60	−41.97	−40.50	−40.89	−42.36	−42.25	−43.97
Δ*E*_GAS_	−69.27	−68.05	−70.18	−167.41	−85.70	−165.50	−60.50
Δ*E*_SOLV_	11.27	5.43	21.10	117.40	13.57	125.95	4.20
Δ*G*_TOT_	−**58.00**	−**62.62**	−**49.08**	−**50.01**	−**72.13**	−**39.54**	−**56.30**

Absolute free energy Δ*G*_TOT_ = (Δ*E*_GAS_ + Δ*E*_SOLV_) − *T*Δ*S*; Δ*E*_GAS_ + Δ*E*_SOLV_ = enthalpy; *T*Δ*S* = solute entropy; Δ*E*_GAS_ = total energy of solute, Δ*E*_GAS_ = Δ*E*_VDW_ + Δ*E*_ELE_; Δ*E*_VDW_ = van der Waal's energy; Δ*E*_ELE_ = electrostatic/coulombic energy; Δ*E*_SOLV_ = total energy of solvation; Δ*E*_SOLV_ = Δ*E*_POL(GB)_ + Δ*E*_NPOL_; Δ*E*_POL(GB)_ = polar solvation contribution, Poisson–Boltzmann method; Δ*E*_NPOL_ = nonpolar contribution.

**Table 4:** Calculated entropy contributions by quasi-harmonic approximation for PPAR gamma-TKI's and PPAR gamma-rosiglitazone complexes by MM-GBSA.

Entropy	PPAR gamma-rosiglitazone	PPAR gamma-ibrutinib	PPAR gamma-sorafenib	PPAR gamma-sunitinib	PPAR gamma-erlotinib	PPAR gamma-gefitinib	PPAR gamma-dabrafenib
Δ*S*_TRANS_	−12.9549	−13.1383	−13.1854	−13.0527	−13.039	−13.1529	−13.2826
Δ*S*_ROT_	−10.8568	−11.2006	−11.426	−11.0008	−11.07	−11.129	−11.2829
Δ*S*_VIB_	−16.9853	−20.0619	−21.389	−19.8061	−19.5318	−20.1034	−23.4465
Δ*S*_TOT_	−40.7974	−44.4008	−46.0007	−43.8597	−43.6408	−44.385	−48.012

Entropy contribution (Δ*S*) is determined by quasi-harmonic approximation. Δ*S*_TOT_ = Δ*S*_TRANS_ + Δ*S*_ROT_ + Δ*S*_VIB_; Δ*S*_TRANS_ = translational entropy contribution; Δ*S*_ROT_ = rotational entropy contribution; Δ*S*_VIB_ = vibrational entropy contribution.
